# Network Hamiltonian models reveal pathways to amyloid fibril formation

**DOI:** 10.1038/s41598-020-72260-8

**Published:** 2020-09-24

**Authors:** Yue Yu, Gianmarc Grazioli, Megha H. Unhelkar, Rachel W. Martin, Carter T. Butts

**Affiliations:** 1 Department of Computer Science, University of California, Irvine, CA 92697 USA; 2 Department of Chemistry, San José State University, San Jose, CA 95192 USA; 3 Department of Chemistry, University of California, Irvine, CA 92697 USA; 4 Department of Molecular Biology and Biochemistry, University of California, Irvine, CA 92697 USA; 5 California Institute for Telecommunications and Information Technology, University of California, Irvine, CA 92697 USA; 6 Departments of Sociology, Statistics, and EECS, University of California, Irvine, CA 92697 USA

**Keywords:** Computational biophysics, Protein aggregation, Networks and systems biology, Protein aggregation, Protein aggregation, Self-assembly

## Abstract

Amyloid fibril formation is central to the etiology of a wide range of serious human diseases, such as Alzheimer’s disease and prion diseases. Despite an ever growing collection of amyloid fibril structures found in the Protein Data Bank (PDB) and numerous clinical trials, therapeutic strategies remain elusive. One contributing factor to the lack of progress on this challenging problem is incomplete understanding of the mechanisms by which these locally ordered protein aggregates self-assemble in solution. Many current models of amyloid deposition diseases posit that the most toxic species are oligomers that form either along the pathway to forming fibrils or in competition with their formation, making it even more critical to understand the kinetics of fibrillization. A recently introduced topological model for aggregation based on network Hamiltonians is capable of recapitulating the entire process of amyloid fibril formation, beginning with thousands of free monomers and ending with kinetically accessible and thermodynamically stable amyloid fibril structures. The model can be parameterized to match the five topological classes encompassing all amyloid fibril structures so far discovered in the PDB. This paper introduces a set of network statistical and topological metrics for quantitative analysis and characterization of the fibrillization mechanisms predicted by the network Hamiltonian model. The results not only provide insight into different mechanisms leading to similar fibril structures, but also offer targets for future experimental exploration into the mechanisms by which fibrils form.

## Introduction

Amyloid fibrils are self-assembling protein superstructures held together by non-covalent bonds and exhibiting local ordering characterized by periodicity along a single axis of growth. Amyloid fibril formation has been shown to occur for a wide variety of biologically relevant proteins under an even wider variety of in vivo and in vitro conditions, making it a general stable state of proteins^1,2^. However, the detailed mechanism of amyloid fibril self-assembly remains a fundamental unanswered question in molecular biology, being impossible to reduce to a simple function of primary sequence: not only can different protein sequences form similar fibrils, but in many cases the same protein sequence can assemble into different fibril types depending on environmental conditions^3–6^. Fibril formation pathways and kinetics are also medically important phenomena, with fibril formation implicated in more than 40 different human diseases (including Alzheimer’s disease and type II diabetes), all of which lack curative treatments^2,7–9^.

Protein aggregation is also a well-known nuisance for in vitro protein production, where it poses technical difficulties with significant economic consequences in the biotechnology and pharmaceutical industries. Understanding fibril formation is essential to the development of strategies to control, manipulate or prevent fibril growth. As such, this area of research has attracted significant attention over the last half century. Currently, no experimentally-derived models describe the exact mechanistic details of fibril formation, as the process is innately complex^10,11^, and standard methods for structural elucidation are limited to the study of fully formed fibril structures. The generally accepted hypothesis on fibril formation states that the starting reactant is monomeric protein, with primary nucleation following the “activation” of one or more monomers^10,11^. Nucleation follows a long incubation period known as the lag phase, which can be shortened by seeding^10,11^. After the lag phase begins rapid formation of oligomers, protofibrils, and eventually, fibrils^10,11^. Another phenomenon that has been reported in the literature is the existence of a *critical oligomer concentration (COC)*, below which the time-dependence of fibril formation exhibits a lag phase with sigmoidal functional form (measured via thioflavin T, or ThT, binding assays), and above which fibril growth occurs with a lag-free initial growth phase^12–14^.

This paper offers a detailed characterization of the dynamics of a recently introduced model for aggregation that recapitulates the self-assembly of all amyloid fibril topologies currently found in the Protein Data Bank (PDB)^15^. The methodology employs *exponential-family random graph models* (ERGMs)^16–18^ to build network Hamiltonian-based statistical models of the formation of experimentally-observed fibril topologies derived from experimentally resolved atomistic fibril structures via solvation energy approximations, using a similar approach to standard solvation energy-based drug docking techniques^15^. Our topological coarse-grained model of fibril self-assembly exhibits extraordinarily high computational efficiency, which we leverage to build models capable of recapitulating the full fibrillization process - from free monomers to fully assembled fibrils - for systems comprising thousands of protein monomers. Here we analyze models for all five of the amyloid fibril topologies found in the PDB to date.

We proceed by first giving a brief overview of modeling amyloid fibril formation dynamics governed by a network Hamiltonian. Next, we describe our approach to using network statistical properties to define a set of *epochs* that are used to describe the reproducible characteristics that define the phases of self-assembly that comprise the mechanism of fibrillization under a given model. A set of such metrics is then used to compare two parameterizations of the network Hamiltonian (corresponding to different experimental conditions) that converge to a commonly observed class of fibril topologies (the *1-ribbon*) but proceed via distinct kinetic pathways. Further analysis on this same pair of models is then carried out by monitoring the appearance and persistence of various types of topologically defined fibril defects. Next, we characterize the epochs of fibrillization for all five currently known fibril topologies in the PDB and conclude with an overview of some of the hallmarks of the fibrillization mechanisms predicted by our models, which we offer as potential targets for future experimental studies.

## Background

**Figure 1:**
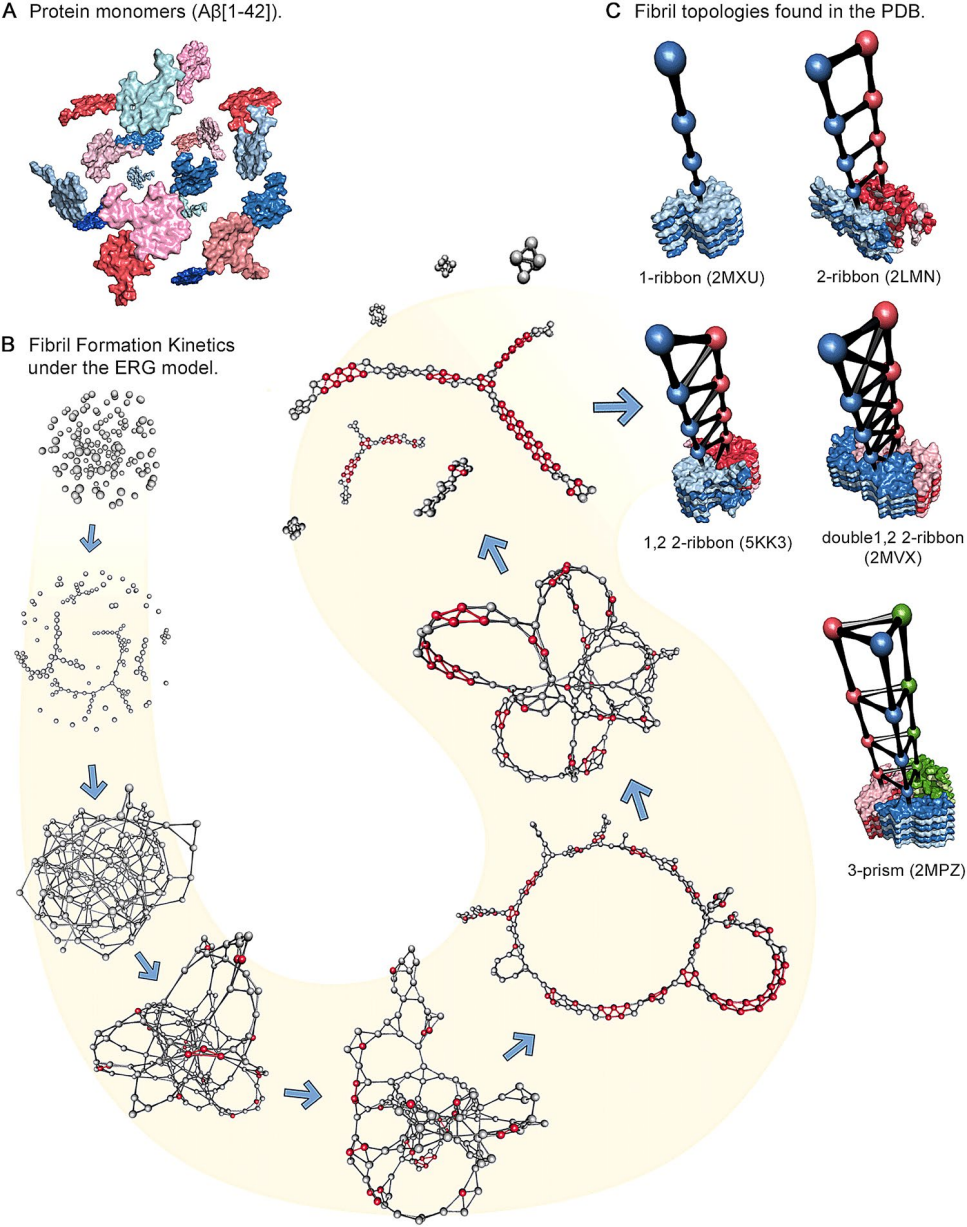
Schematic depiction of the aggregation graph representation of fibril formation. (**A**) Free protein monomers in solution. Each monomer becomes mapped to a vertex in the aggregation graph. (**B**) Network visualization of the fibrillization process, as it occurs under our model, using the 1,2 2-ribbon as an example. We begin with an empty graph corresponding to a population of free monomers, which passes through various intermediate states *en route* to fibril formation. (**C**) Examples illustrating all five experimentally observed fibril topologies currently observed in the PDB. Structures are from PDBIDs 2MXU^19^, 2LMN^3^, 5KK3^20^, 2MVX^21^, and 2MPZ^22^; each can be characterized in terms of a repeating pattern of connectivity among monomers.

Our modeling framework is based on the notion of *aggregation graphs*, topological representations of protein aggregation states in which each vertex corresponds to a single protein monomer, and each edge corresponds to a non-covalent interaction between monomers. Figure 1 provides an overview of the use of aggregation graphs in the context of fibril formation. Protein monomers (Fig. 1A) are mapped to vertices in the aggregation graph; for free monomers in solution, the initial system state corresponds to an empty graph. As the fibrillization process proceeds, the topology of connections among monomers evolves (Fig. 1B), eventually leading to a characteristic fibril topology that comes to dominate the system in equilibrium. Recent work^15^ has found that all amyloid fibril structures so far found within the PDB correspond to five topological classes (as shown in Fig. 1C); in graph-theoretic nomenclature, these are described as (respectively) 1-ribbons; 2-ribbons, 1,2 2-ribbons, double 1,2 2-ribbons, and 3-prisms. (See Supplemental Section S1 for a summary of topological nomenclature.) The aggregation graph formalism provides a coarse-grained representation of aggregation states that is highly efficient while still retaining considerable flexibility in describing distinct patterns of intermolecular interaction.

The evolution of an aggregation graph is governed by a topological Hamiltonian, a coarse-grained energy function whose terms are defined with respect to topological degrees of freedom of the protein system. Although this approach can be used to model a wide range of both structured and unstructured aggregates, prior work in the context of amyloid fibrils has shown^15^ that the five topologies described above can be recapitulated by a Hamiltonian of the form:

1 $$\begin{aligned} \begin{aligned} {\mathcal {H}}(g)&=\left( \phi _e + k_B T \right) t_e(g) + \phi _{2s} t_{2s}(g) + \phi _{NSP1} t_{NSP1}(g) \\&\quad + \phi _{NSP2} t_{NSP2}(g) + \phi _{ESP0} t_{ESP0}(g) + \phi _{ESP1} t_{ESP1}(g) \\&\quad + \phi _{C5} t_{C5}(g) + \phi _{C6} t_{C6}(g) + \phi _{C7} t_{C7}(g) \end{aligned} \end{aligned}$$

where *g* is the graph representing the non-covalent bonding structure between monomers, $k_B$ is Boltzmann’s constant, *T* is temperature, the *t*(*g*) functions return a count of the number of times that a particular bonding motif appears in graph *g*, and the $\phi$ values are the signed coefficients that scale a given bonding motif’s contribution to the enthalpy of the aggregation state. Per previous work^15^, the terms are defined as follows. $t_e(g)$ is the number of edges in *g*, and determines the base energetic cost of an edge in the absence of other interactions. $t_{2s}(g)$ counts the number of *two-stars* in *g*, i.e., configurations involving a monomer bound to two other monomers; intuitively, $t_{2s}(g)$ can be thought of as capturing the first-order effect of each existing bond on the cost of forming additional bonds. $t_{NSP1}(g)$ and $t_{NSP2}(g)$ are *null shared-partner* terms, counting specifically the numbers of unbound monomer pairs having respectively bound to exactly one or two common neighbors. The NSP terms are multi-body interactions that affect the propensity to form straight segments or chordless four-cycles, and can be viewed as related to the deformability of local structures formed by successive series of bound monomers. (A similar physical interpretation applies to terms $t_{C5}$–$t_{C7}$, which respectively count the numbers of cycles of length five, six, and seven; these can be seen as expressing higher-order rigidity effects.) Finally, the *edgewise shared-partner* terms^23^ count the number of bound monomer pairs having respectively no ($t_{ESP0}$) or one ($t_{ESP1}$) common partner in the aggregation graph. These terms are related to triadic closure, i.e., the tendency of monomers with common partners to bind to one another. The energetic contribution of each of these topological degrees of freedom is determined by $\phi$; at present, $\phi$ values are determined empirically based on their ability to reproduce observed fibril topologies in equilibrium. Here, we use the $\phi$ values identified by^15^, as provided in Supplemental Table S1.

For computational purposes, it is often useful to work with a modified representation of the graph Hamiltonian parameterized by parameter vector $\theta =-\phi /(k_B T)$. In equilibrium, an aggregation graph driven by such an energy function will follow a probability distribution given by

2 $$\begin{aligned} \Pr (G=g|\theta ,t) = \frac{\exp \left( \theta ^T t(g)\right) }{\sum _{g' \in {\mathcal {G}}}\exp \left( \theta ^T t(g')\right) h(g')} h(g), \end{aligned}$$

where *G* represents the aggregation graph, *g* is a particular graph microstate drawn from the set of potentially observable graph microstates ${\mathcal {G}}$, and *h* is a *reference measure* corresponding to entropic effects arising from hidden degrees of freedom in the aggregation system. In the network modeling literature, a model of this form is referred to as an exponential-family random graph model (ERGM)^24^; ERGMs have been extensively studied in the context of network statistics^16,25,26^ and social networks^27–29^, where they have been employed to model relational systems with complex dependence among edges. From the view of statistical mechanics, Eq. (2) is immediately recognizable as being a Boltzmann distribution on ${\mathcal {G}}$, with the exponent $-{\mathcal {H}}(g)/(k_BT) = \theta ^T t(g)$ and $\log h(g)$ representing the entropy of *g* (the latter taking a unit value when *h* is the counting measure on ${\mathcal {G}}$). An advantage of working with with the ERGM representation of *G* is that simulation methods for such distributions are fairly well-studied (e.g.^30–32^) and standard Markov chain Monte Carlo (MCMC) implementations are available. In addition to MCMC techniques for sampling from the thermodynamic distribution implied by a given network Hamiltonian, it is also possible to carry out kinetic sampling using these models to produce time-dependent trajectories of amyloid fibril aggregation graphs initiated from empty graphs (zero bonds shared between monomers). All simulations in the present work were carried out using the latter methodology, i.e. the kinetic extension of the fibrillization ERGM^15^, which is described in the “Methods” section.

## Results

As noted above, an overview of our topological simulation methodology and classification scheme is shown schematically in Fig. 1. All aggregation simulations begin with a set of free monomers. As the simulation proceeds, free monomers start to bond to each other, forming larger (typically unstructured) aggregates. Next, locally fibrillar protein monomers (i.e. monomers sharing bonds with immediate neighbors in accordance with a fibrillar topology) begin to form at different locations within the aggregates. As new fibrillar regions form and join with the existing fibrillar content, the local fibrillar structures grow both in quantity and size. While larger fibrillar subunits appear in multiple locations of the aggregate, oligomers (in graph theoretic terms, components of size less than 8) also form. Details of the aggregation process for each of the pathways observed in these simulations are given in Supplementary Figs. S1–S4. Surprisingly, the 1-ribbon, the simplest and one of the most common fibril topologies, can be produced via two different mechanisms. This result has relevance for experimental studies of fibrillization kinetics, and presents a possible mechanism for how more or less toxic oligomers can be formed along the pathways to similar fibrils.

**Figure 2:**
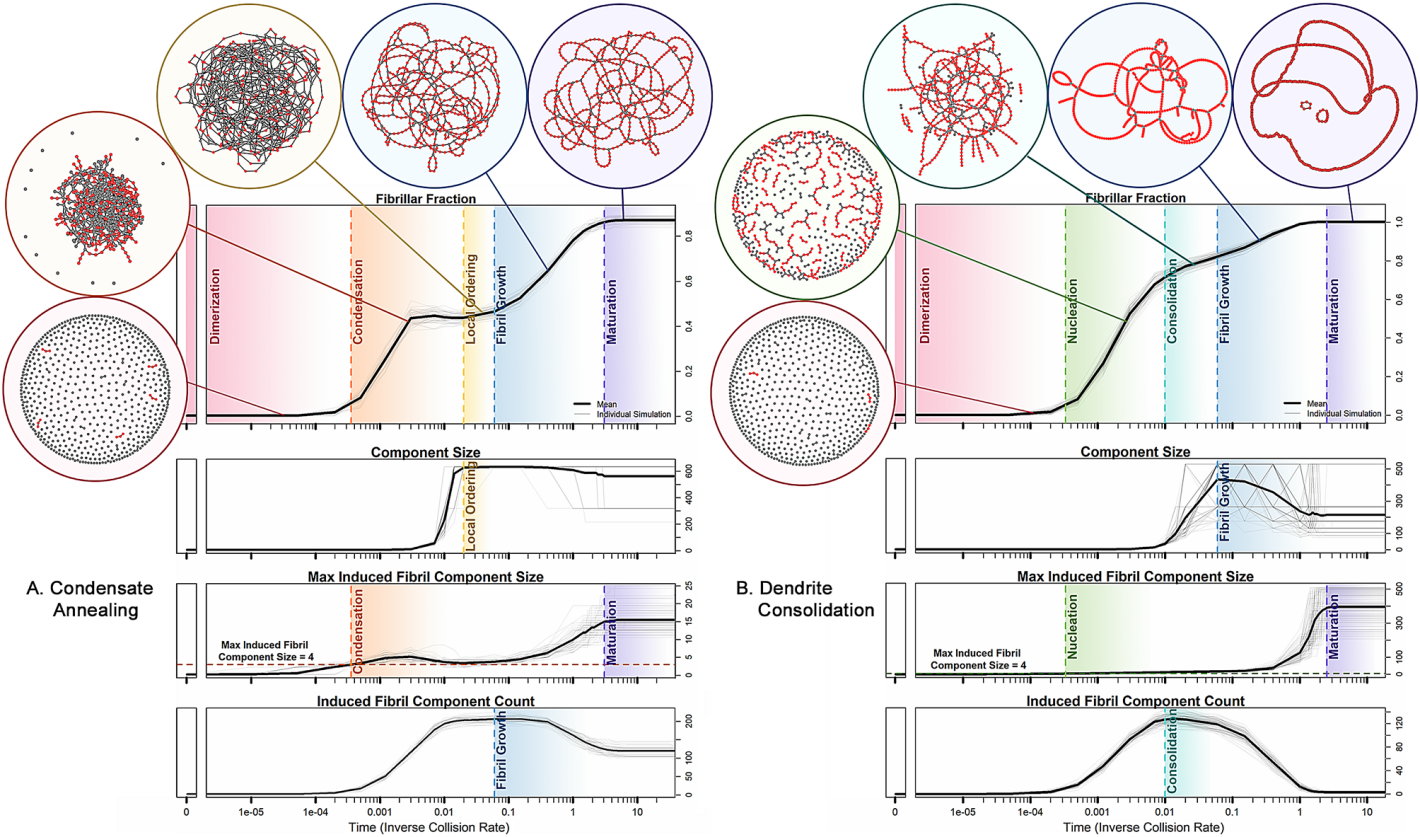
Two different pathways to fibril formation. (**A**) and (**B**) illustrate the epochs under processes associated with two different 1-ribbon parameter values, which we call *condensate annealing* and *dendrite consolidation*, respectively. Here we demonstrate that different parameterizations of our model are able to capture rich dynamics for even the simplest fibril topology, the 1-ribbon. Although the initial dimerization epoch is similar for both parameters, the subsequent dynamics are quite different between the two models. Under the condensate annealing model (**A**), the protein monomers quickly reach a disordered condensate state, where the proteins form a large connected component. After that, defects anneal out, and fibril components connected by branches emerge. Under the dendrite consolidation model (**B**), there is no condensate epoch. Instead, fibrils grow from sparsely connected components, where the average size of the components grows as fibril size grows, resulting in long, pure fibrils.

Observation of the detailed kinetics of fibril formation across different fibril types reveals the presence of distinct periods characterized by the topology of the evolving aggregation graph, which we refer to here as “epochs.” The measures used to define the set of epochs describing the mechanism of fibril formation under a given model are displayed in Fig. 2 (an illustrative example of these network measures is also given in Fig. S5 in the Supplement). Here, we illustrate the formation of 1-ribbons from monomers via two different pathways determined by the initial parameters ($\phi$). Average component size refers to the mean number of protein monomers that comprise each aggregate (component) in a given aggregation state. The second and third measures are based on the concept of *induced fibrillar component*, which is adapted from general graph theory. In graph theoretic terms, an induced subgraph is a graph that is formed by some subset of nodes from a larger graph, together with all edges connecting those nodes. A (maximal) component is a maximal connected subgraph. Applying this graph theoretic construct to networks describing protein aggregates, an induced fibrillar component is then a set maximal of monomers that are (1) in a locally fibrillar conformation, and (2) that are joined to one another by a series of non-covalent bonds. For example, the rightmost aggregate shown in Fig. S5 in the Supplementary Information contains a 2-ribbon induced fibrillar component of length 5. Since fibril topology is, by definition, periodic, at least two adjacent minimal repeating subunits must lie within a subgraph for it to be considered an induced fibrillar component. The *maximum induced fibril component size* measure is then defined as the number of protein monomers that comprise the largest fibrillar component within the aggregation structure. Likewise, the *induced fibril component count* measure is defined as the number of distinct fibrillar components present in the aggregation state. Together, these two measures allow us to track different aspects of the fibrillization process. For example, note the way that the induced fibril component count drops precipitously in the late phase of the dendrite consolidation model (Fig. 2B, bottom panel), due to the absorption of smaller fragments into large, pure, fibrils, while the same measure for the condensate annealing model converges to a non-zero value (Fig. 2A, bottom panel), due to the formation of many smaller fibril components that attach to each other via non-fibrillar components we call *defects*. These measures form the basis for characterizing the epochs of fibril formation.

**Figure 3:**
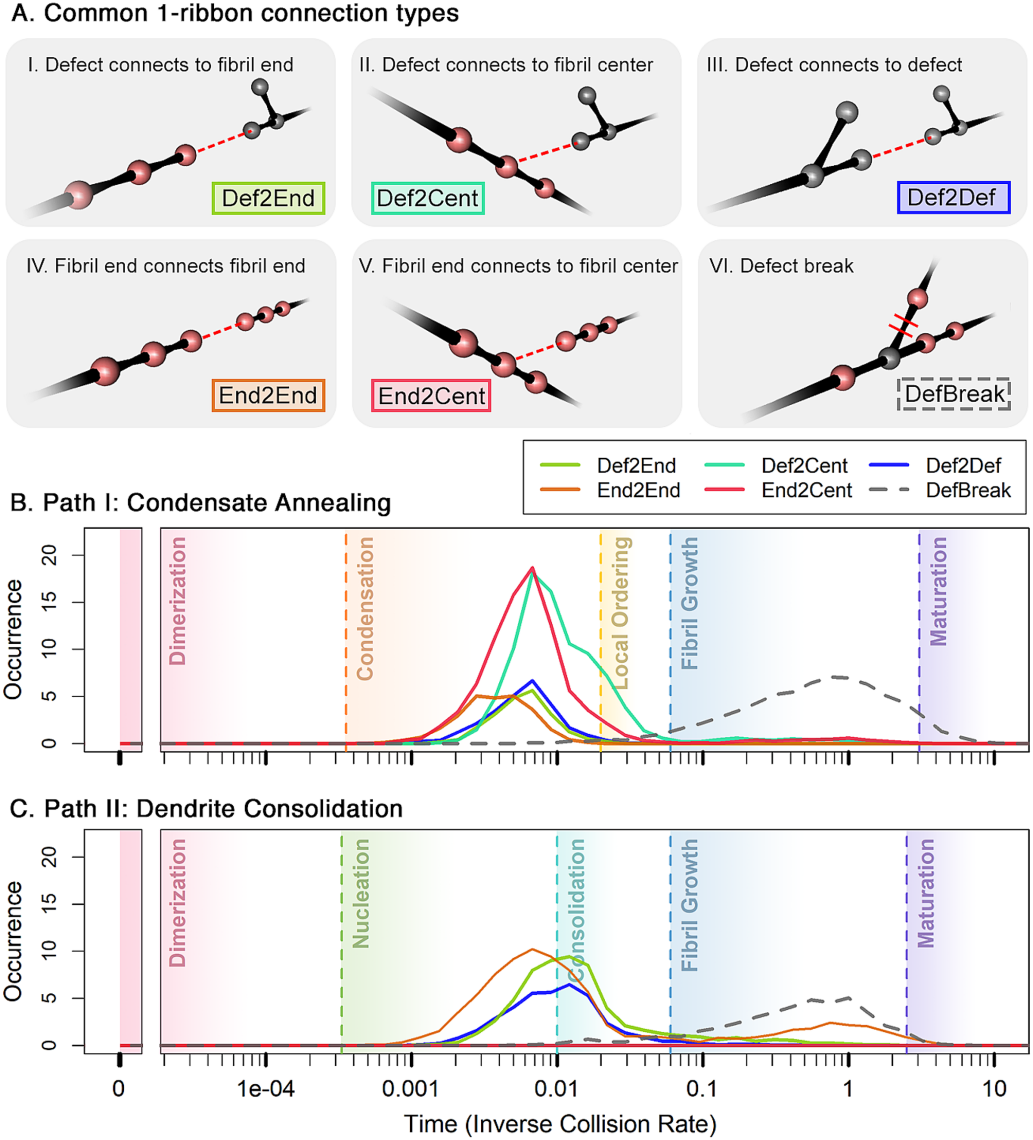
Illustration of different intermolecular bonding motifs during 1-ribbon formation. (**A**) Illustrates all observed bonding motifs. (**B**) and (**C**) present a summary of the occurrence of each bonding motif under the condensate annealing and dendrite consolidation models, respectively. The most significant difference is that under the condensate annealing model, there are many instances where a defect is connected to the fibril center (Panel A*II*), whereas, under the dendrite consolidation model, this type of connection is much rarer. Another significant difference is that the condensate annealing model also produces a significant number of connections from fibril ends to fibril centers (Panel A*V*), whereas this bonding motif is almost non-existent under the dendrite consolidation model. Although both models produce substantial fibril yield in the experimentally observed 1-ribbon topology, the fibril yield and purity are superior in the dendrite consolidation model.

**Figure 4:**
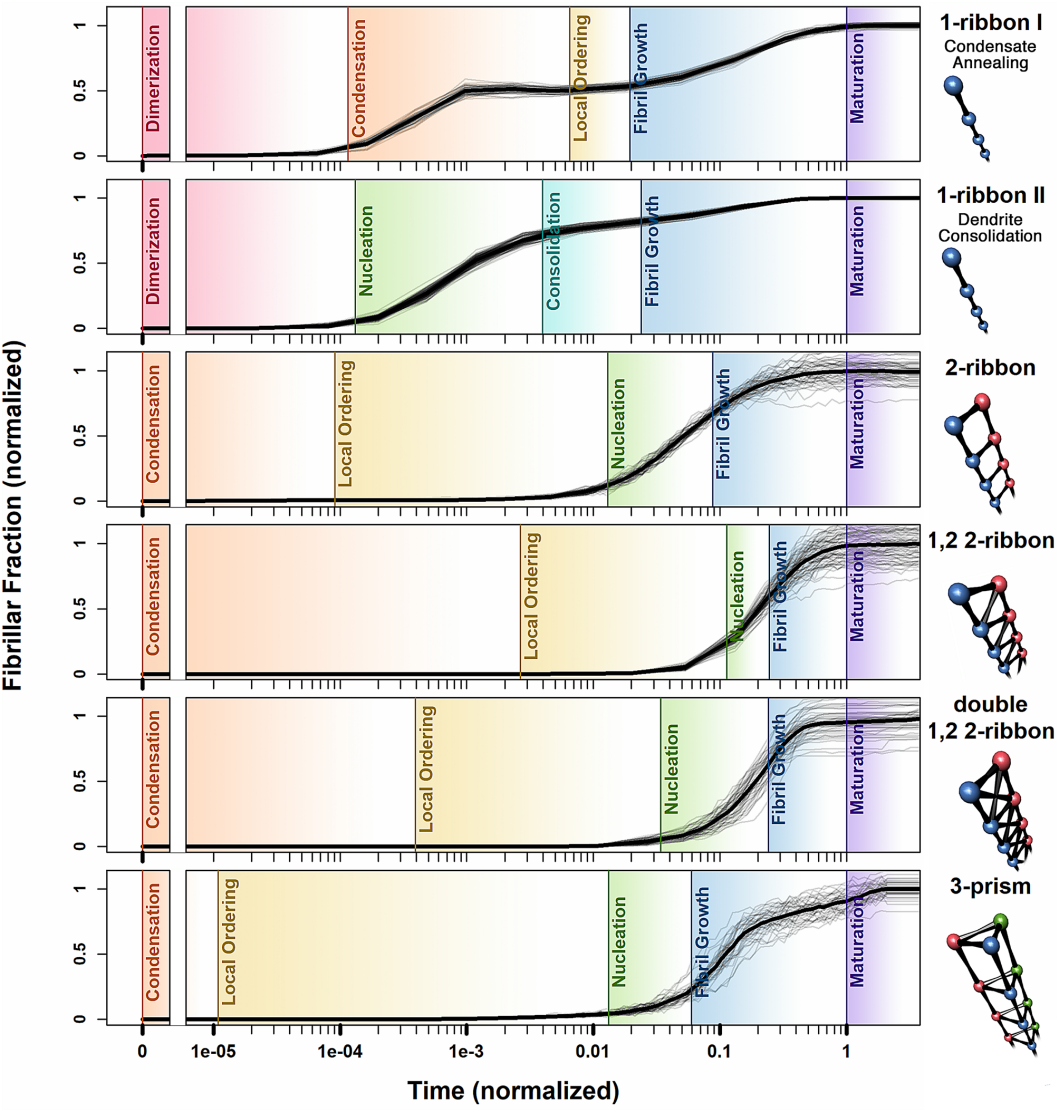
Overview illustrating the different epochs observed for all five experimentally observed fibril topologies, including both models for 1-ribbon formation. Immediately notable is the stark contrast between the epochs characterizing 1-ribbon growth vs. the topologies exhibiting higher-order structure. It is noteworthy that although the two models for 1-ribbon growth present different mechanisms for fibril self-assembly, both models exhibit a gentler fibrillar fraction curve of steady growth compared to the more complex topologies, which exhibit a prefibrillization lag phase followed by a sharp increase in fibril formation. This result offers a target for future experimental probes into characterization of previously unobserved latent self-organization events prior to fibril formation.

A comparison among the epochs that characterize the formation of each fibril type is shown in Fig. 4. We note that the sharpest contrast that can be made from this information is the difference between the two 1-ribbon models and all other fibril topologies (higher order structures). Although both 1-ribbon models are characterized by steady, gradual fibril growth, the higher-order topologies all exhibit a common initial lag phase, followed by a sharp increase in fibril fraction. It is also noteworthy that the fibrillization dynamics of the four higher-order fibril topologies can be characterized using the same set of epoch definitions; the differences between types are in the relative epoch lengths. All simulations begin with an average component size of one, because all proteins are monomeric, i.e. all components comprise a single protein molecule. As more bonds form, the average component size increases until it reaches a maximum. We mark the period from the beginning of the simulation to this point as the *Condensation* epoch. The next epoch, *Local Ordering*, is characterized by formation of short fibrillar structures embedded sparsely in an otherwise unstructured protein aggregate. These short stretches of fibril then grow longer as more fibrillar subunits attach to the end. When the length of the longest fibrillar aggregate, i.e. *maximum induced component size*, reaches a certain threshold, we start to see clear fibril structures embedded in the larger protein aggregate; and this marks the start of the *Nucleation* epoch. The threshold is chosen such that at least two full minimal repeating subunits appear. For example, for the 3-prism, where the minimal repeating subunit is a triangular component with three monomers per subunit, the threshold is chosen to be 6 protein monomers, representing a fibrillar component with two full minimal repeating subunits. The only exception to this rule of a threshold defined by two repeating subunits is the 1-ribbon, for which we set the threshold to four, as a two subunit cutoff would result in all instances of a pair of protein monomers sharing an intermolecular bond being classified as fibrillar.

During the Nucleation epoch, more short stretches of fibril emerge from the unstructured protein aggregate, which results in the induced fibril component count continuing to increase. When the number of induced fibril aggregates reaches a maximum, we mark it as the start of the *Fibril Growth* epoch. After a large fraction of the protein monomers are bound within fibrils, increase in the fibril fraction tends to occur by connecting existing fibril subunits instead of forming new isolated fibrils. While this consolidation phenomenon increases the induced fibril component size, it decreases the induced component count. For example, given a system comprising two fibrils of equal length, if the two fibrils bind together, this would simultaneously double the induced fibril component size and reduce the induced component count from 2 to 1. The last stage (*Maturation*) is when both the fibril fraction (fraction of total proteins bound within a fibril) and the max fibril component size (number of proteins comprising the largest fibril in the system) appear to converge to some equilibrium value. The progression of epochs that emerges from network Hamiltonian simulations - from Condensation to Local Ordering to Nucleation to Fibril Growth and finally Maturation - is in agreement with prior works that indicate a similar progression in unseeded fibril formation dynamics: from a lag phase in ThT fluorescence with prolific nucleation^33^, to the formation of on-pathway oligomers^12^ (compare with the nucleation epoch in the Dendrite Consolidation model), to other events, such as fragmentation with secondary nucleation (compare with defect breakage and defect to end connections), and elongation (end to end connections)^34–36^. Furthermore, the appearance of a large, pre-fibrillar component during the condensation phase of 1-ribbon formation via the condensate annealing mechanism is consistent with the transparent hydrogels observed during the fibrillization of superoxide dismutase^37^ and $\beta$-lactoglobulin^38^. Such similarities are further addressed in the “Discussion” section.

## Discussion

Despite the scientific and medical importance of understanding amyloid fibril formation, detailed mechanisms of fibrillization have remained elusive. Complications that have impeded the elucidation of the mechanism of fibrillization include reproducibility challenges like substantially varying fibril growth lag times in apparently identical experiments^11,39^, the selection bias inherent to experiments used to measure periodic structure (i.e. defects are either purified out during sample preparation or averaged out during measurement), and fibrillization timescales that lie many orders of magnitude beyond the reach of standard molecular simulations. The development of network Hamiltonian models for aggregation^15^, along with the methods presented for quantitatively characterizing the kinetic pathways to amyloid fibril formation predicted by these models, offer suggestions for future experimental probes into the mechanisms of fibril formation. For example, all network Hamiltonian models examined here for fibril formation of topologies exhibiting more complexity than the 1-ribbon enter into an initial condensate phase. This is noteworthy, as it implies formation of a disordered aggregate as a precursor to fibril formation, which is in direct contrast to models of fibril formation where monomers transition directly from free monomer status to binding to the growth face of a fibril. It has been widely observed that fibril-forming solutions often enter into a gel-like and sometimes high-turbidity state prior to fibril formation^5,40,41^. Bearing in mind limitations on locality imposed by the rate of diffusion, this gelatinous and/or turbid state would be consistent with the formation of droplets of the disordered condensate predicted by the network Hamiltonian models.

Established chemical kinetics models of fibril formation have repeatedly demonstrated the importance of including rate laws that account for fragmentation and secondary nucleation^34–36^. Thus, it is interesting to observe that both fragmentation and secondary nucleation events have been observed in our models as emergent properties from the parameterization of the terms in the network Hamiltonian. For example, the 1-ribbon results shown in Fig. 2 suggest a mechanism of fibril growth whereby secondary nucleation is followed by fragmentation. The effect is particularly pronounced in the consolidation and fibril growth phases of the dendrite consolidation (Fig. 2B). Additional evidence of a fragmentation-driven fibril growth phase in the dendrite consolidation model is demonstrated quantitatively in Fig. 3, where a clear second wave of end-to-end growth coincides with peak defect breakage. Other kinetic models for fibril formation have established the importance of accounting for both initial attachment of monomers to the free end of an existing fibril and the subsequent rearrangement that leads to the newly attached monomers maturing into part of the fibril component, a notable example being the Petri net introduced by Meisl et al. (2017)^42^, where such effects are explicitly added to the model. This is consistent with multiple emergent phenomena observed in our models, including the manner in which they progress from consolidation, to fibril growth, to maturation. Another interesting feature in the behavior of our models is the consistent appearance of a condensation epoch, a phase characterized by a highly connected and disordered aggregate, that occurs during the lag phase of fibril growth for many fibril types. This is consistent with prior work^33^ suggesting that the lag phase is not merely a period of waiting for nucleation to happen, but rather a period of prolific nucleation. For instance, Arosio et al.^33^ posit that the lag period is due to a delay in fibrillar aggregates reaching a detectable size, and that this is consistent with the pattern of lag phase followed by exponential growth of fibril yield consistently observed in unseeded fibril growth assays. Our models consistently display the lag phase, growth or elongation phase, and saturation phases that are well-known and expected of models throughout the literature^43^, with the added feature that this behavior spontaneously emerges from the action of a low-dimensional network Hamiltonian.

Another area where network Hamiltonian models have the potential to guide additional experimental studies is in the realm of fitting experimental fibrillization kinetics assay data to fibril fraction curves produced by network Hamiltonian aggregation models. There exists a wealth of uncharted territory when it comes to describing the intermolecular interactions that lead to amyloid fibril formation, and a paucity of models capable of capturing the entire process of thousands of free monomers self-assembling into fully formed, thermodynamically stable, and kinetically accessible amyloid fibril structures. Network statistical characterization of the pathways to fibrillization predicted by network Hamiltonian models offer a uniquely capable approach toward identifying potential intermediates in the process of amyloid fibril formation for experimental validation or invalidation.

## Methods

The kinetic extension of the ERGM framework, used here to model time-dependent aggregation events, is obtained by adapting a common model of reaction kinetics to accommodate a large number of kinetic pathways. Before addressing the many-pathway treatment, let us begin with a simple two state system, where we are interested in transitions between states *i* and *j*, and for convenience, we define $\beta =1/(k_b T)$, ${\mathcal {H}}_j={\mathcal {H}}(j)$ and $s_j=\log h(j)$. If we assume Boltzmann statistics at equilibrium, where the time independent probability of finding our system in state *j* is $P_j \propto \exp (-\beta {\mathcal {H}}_j + s_j)$, then the conditional probability that the system is in state *j*, given that it is in either state *i* or *j* is:

$$\begin{aligned} \frac{P_j}{P_i+P_j}&= \frac{1}{ 1 + \exp \left[ \beta \left( {\mathcal {H}}_j-{\mathcal {H}}_i\right) +s_i-s_j\right] }\\&=\left[ 1+\exp \left[ \beta \Delta ^{{\mathcal {H}}}_{ij} - \Delta ^S_{ij}\right] \right] ^{-1} \end{aligned}$$

where $\Delta ^{{\mathcal {H}}}_{ij}$ represents the difference in energy going from state *i* to state *j*, and $\Delta ^S_{ij}$ represents the corresponding difference in the log reference measure (microstate entropy). From this unitless expression, we can obtain an equation for the conditional rate of formation of state *j* by multiplying by some event frequency *A* of units events per time (e.g. a collision rate):

3 $$\begin{aligned} r_{ij} = \frac{A}{1+\exp \left[ \beta \Delta ^{{\mathcal {H}}}_{ij} - \Delta ^S_{ij}\right] }. \end{aligned}$$

Note that if $\Delta ^{{\mathcal {H}}}_{ij}$ is treated as an activation energy barrier $E_a$ in going from state *i* to *j*, the barrier is both “uphill,” i.e. positive, $\exp \left[ \beta \Delta ^{{\mathcal {H}}}_{ij} - \Delta ^S_{ij}\right] \gg 1$, and $\Delta ^S_{ij} \approx 0$, Eq. (3) reduces to the familiar Arrhenius law for calculating chemical rate constants:

$$\begin{aligned} k_{ij} = A e^{-\beta E_a} \end{aligned}$$

while a “downhill” change for $\beta \Delta ^{{\mathcal {H}}}_{ij} - \Delta ^S_{ij}$, i.e. a negative free energy difference, leads to the rate of *j* formation in Eq. (3) to approach the collision frequency, thus embedding spatial limitations due to dynamics into a non-spatial model.

Next, we generalize the approach to a large number of potential reaction pathways. In the model, all changes to the aggregation graph are presumed to occur in series, a weak constraint given the rarity of true simultaneity in the limit of arbitrary time precision. Thus, any transition from state *i* to *j* is possible if and only if *i* and *j* differ by exactly one bond (a single Hamming step). Moreover, if the graph is currently in state *i*, all allowed $i\rightarrow j$ transitions represent the competing reactions. Following Eq. (3), if ${\mathcal {N}}_i$ represents the set of aggregation graphs one Hamming step away from *i*, then it follows that

$$\begin{aligned} R_{i+} = \sum _{j \in {\mathcal {N}}_i} r_{ij} \end{aligned}$$

is the exit rate for state *i* and

$$\begin{aligned} P_{ij} = r_{ij}/R_{i+} \end{aligned}$$

is the probability that the next realized transition is from state *i* to state *j*, given that the system is currently in state *i*. Thus, the time to transition out of state *i* will be exponentially distributed with expectation $1/R_{i+}$, which provides the basis for a straightforward simulation algorithm^15^. An interesting observation is that our simulation methodology can be interpreted as modeling fibril growth via time-dependent stochastic dynamics governed by a potential energy surface, a notion which has also been suggested by Buell et al. (2010)^44^.

It is worthwhile to include a brief justification for our use of inverse collision rate as a measure of simulation time. This approach contributes to the rich history in physical chemistry of relating theoretical models for chemical kinetics to latent microscopic degrees of freedom like collision rates between molecules and the fraction of such collisions that are geometrically favored to be reactive. Such approaches have been applied as far back as the foundational work by Eyring^45^ and Arrhenius^46^, and more recently toward fitting traditional chemical kinetics models to experimentally measured amyloid-$\beta$ fibrillization kinetics data^47^. In all of these treatments, our network Hamiltonian approach included, it is necessary to fit the functional form produced by the model to some constant scaling in time that is dictated by latent molecular degrees of freedom. Rather than scale the time dependence of these fibrillization models to particular frequency scaling factors fit to specific experiments (as carried out in the article introducing our simulation scheme^15^), here we report fibril formation as a function of time on the time scale of inverse collision rate. This more general treatment of time-dependence allows us to report fibrillization in a manner that leaves the data in a form that is a simple multiplicative scaling of the *x*-axis away from fitting to any specific experimentally measured reaction kinetics data.

A detailed description of the network Hamiltonian formalism and accompanying simulation methodologies can be found in the work detailing the initial development and implementation of the network Hamiltonian framework^15^, and all model parameters for carrying out simulations using the models included in the present work are given below in Table S1 in the Supplementary Information. Additionally, Figs. S1, S2, S3, and S4 in the Supplementary Information show the epochs of fibril growth for the 2-ribbon, 1,2 2-ribbon, double 1,2 2-ribbon, and 3-prism, respectively. The network measures used to construct the fibril growth epochs, which were used to characterize the fibril formation pathways are demonstrated in Fig. S5. The demonstration is intended to aid intuition for the measures by applying them to a network small enough that the measures can be easily counted. An approach to parameterizing network Hamiltonian models has also been described by Yu et al. (2019)^48^. All simulations and analyses were performed via custom scripts in the R statistical computing environment^49^, using the statnet library^17,50,51^ and the ergm.graphlets package^52^.

## Supplementary information

Supplementary information.
