# Supplementary material for: Network Hamiltonian models reveal pathways to amyloid fibril formation
Source: Sci Rep. 2020 Sep 24;10:15668. doi: 10.1038/s41598-020-72260-8 (PMC7515878; doi:10.1038/s41598-020-72260-8)
Supplement: Supplementary file 1 — Supplementary information. [file 41598_2020_72260_MOESM1_ESM.pdf]

Supplementary Information for "Network Hamiltonian Models Reveal Pathways to Amyloid Fibril Formation"

Yue Yu[1], Gianmarc Grazioli[2], Megha H. Unhelkar[3], Rachel W. Martin[3,4], and Carter T. Butts[1,5,6,*]

[1] *Department of Computer Science, University of California, Irvine, CA 92697*
[2] *Department of Chemistry, San José State University, San Jose, CA 95192*
[3] *Department of Chemistry, University of California, Irvine, CA 92697*
[4] *Department of Molecular Biology and Biochemistry*
University of California, Irvine, CA 92697
[5] *California Institute for Telecommunications and Information Technology*
University of California, Irvine, CA 92697
[6] *Departments of Sociology, Statistics, and EECS*
University of California, Irvine, CA 92697
[*] Corresponding author: *buttsc@uci.edu* , *+1 (949) 824-8591*

As supplementary information for this paper, we include the following materials:

- Overview of Fibril Topology Nomenclature
- Table of ERGM Parameters (Table 1)
- Characteristic Aggregation Dynamics for the 2-ribbon (Figure S1)
- Characteristic Aggregation Dynamics for the 1,2 2-ribbon (Figure S2)
- Characteristic Aggregation Dynamics for the double 1,2 2-ribbon (Figure S3)
- Characteristic Aggregation Dynamics for the 3-prism (Figure S4)
- Heuristic for Network Measures (Figure S5)

S1: Overview of Fibril Topology Nomenclature

In addition to the present overview of the naming convention for intermolecular fibril topology, an in-depth description of the set of rules that comprise a generalizable fibril topology nomenclature that encompasses all currently known fibril topologies can be found in prior work by Grazioli et al. [1] The nomenclature is defined in such a way as to present a concise mathematical description of connectivity between molecules in any aggregate state including but not limited to: protein monomers, small oligomers, larger unstructured aggregates, and the repeating units of connectivity exhibited by fibrils. Graph theory is the natural choice for both the mathematical foundation and terminology needed describe aggregates of molecules in terms of their topology. In particular, exponential-family random graph models (ERGMs), often used to model systems ranging from social networks to systems of neurons [2], offer an ideal framework. In aggregation graph models of this type, each *node* or *vertex* of the graph is an entire protein molecule, and each *edge* or *tie* represents a non-covalent bond between those two molecules. Essential to the system of fibril topology nomenclature is identifying the minimal repeating subunit that characterizes a particular fibril type. For example, a single polymer chain of monomers, where each interior monomer shares a bond with only its neighbors before and after that monomer along the fibril growth axis, is known as a *1-ribbon*. Similarly, the term *2-ribbon* defines a fibril topology where the minimal repeating unit is a pair of monomers, as shown in figure S5. The nomenclature can also be extended to annular minimal repeating subunits, known as *prisms*, e.g. the *3-prism* shown in Figure 1 of the main text. The nomenclature can also be extended to accommodate the addition of further ties, or *chords*, to these fundamental types of repeating units.

An example of applying the chording rules of this system of nomenclature is the *1,2 2-ribbon* shown in Figure S5. In the case of the 1,2 2-ribbon, the 2-ribbon topology has been augmented with the addition of chords that stretch from each subunit to the one exactly one subunit away, i.e. from index 1 to index 2 along the fibril axis. For further detail on the fibril topology nomenclature, the reader is directed to prior work by Grazioli et al. [1]

	1-ribbon Condensate Annealing	1-ribbon Dendrite Consolidation	2-ribbon	1,2 2-ribbon	double 1,2 2-ribbon	3-prism
ERGM Parameters						
edges	115.12	80.26	102.12	158.01	481.46	178.52
kstar(2)	-32.00	-55.14	-25.00	-25.55	-64.26	-36.94
nsp(1)	-1.00	22.61	-1.00	-8.46	0.12	5.58
nsp(2)	-	-	3.00	-2.23	25.31	15.73
esp(0)	-	-	-	-20.96	-77.35	2.64
esp(1)	-	-	-	-	-65.40	11.36
semicycle(5)	-	-	-	-	-	-0.10
semicycle(6)	-	-	-	-	-	-0.30
semicycle(7)	-	-	-	-	-	-0.25
Simulation Time	20	20	700	90	750	4000
Network Size			600			
# of Replicates			100			

Table 1: Detailed parameters for all ERGM simulations carried out for this work. The simulation times shown in this table reflect a nominal simulation time (in units of inverse collision rate) spanning each simulation, during which the output graph was sampled at an exponential increment. Simulation times vary, as some fibrillization models require more time to converge to their respective steady state, and further simulation of the faster converging models provide no additional information. The results were averaged over 100 simulations.

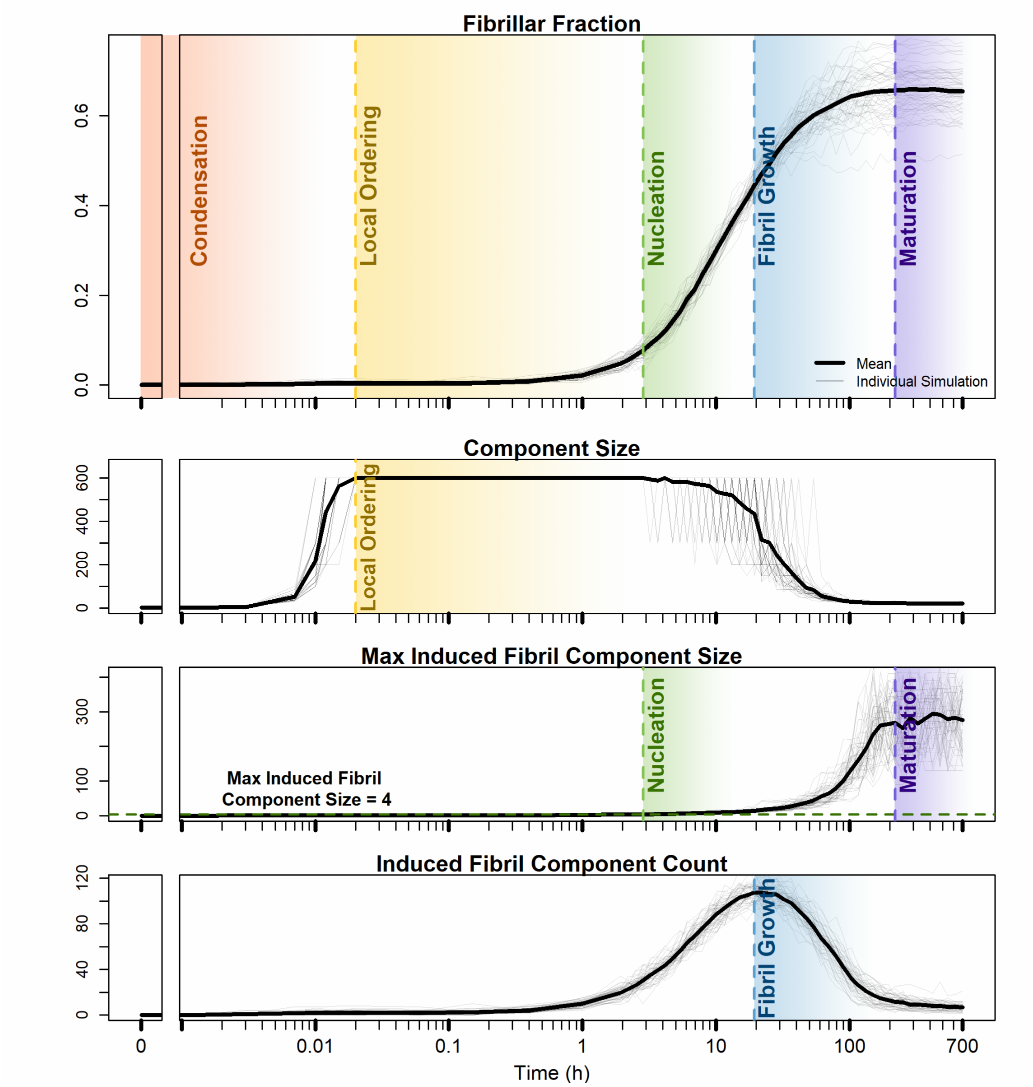

Figure S1: Characteristic aggregation dynamics for the 2-ribbon amyloid fibril network topology.

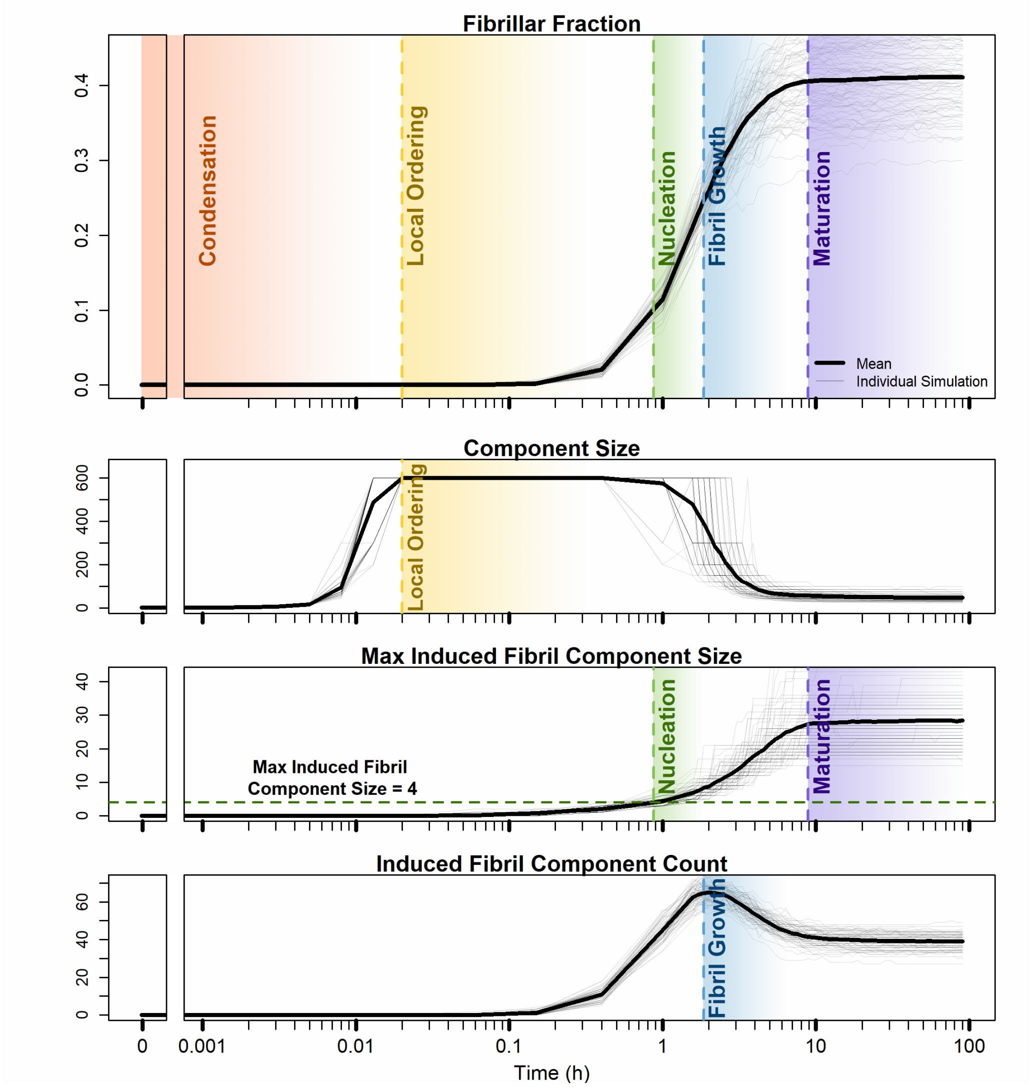

Figure S2: Characteristic aggregation dynamics for the 1,2 2-ribbon amyloid fibril network topology.

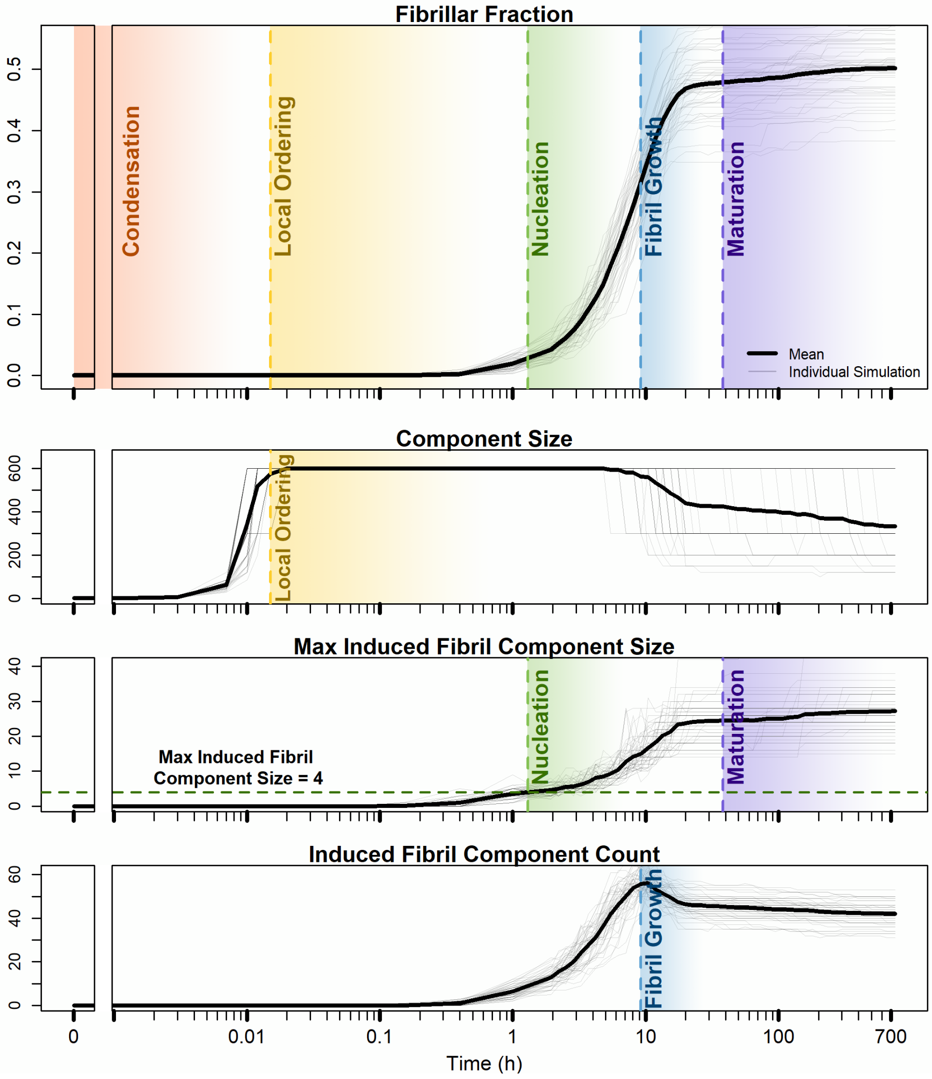

Figure S3: Characteristic aggregation dynamics for the double 1,2 2-ribbon amyloid fibril network topology.

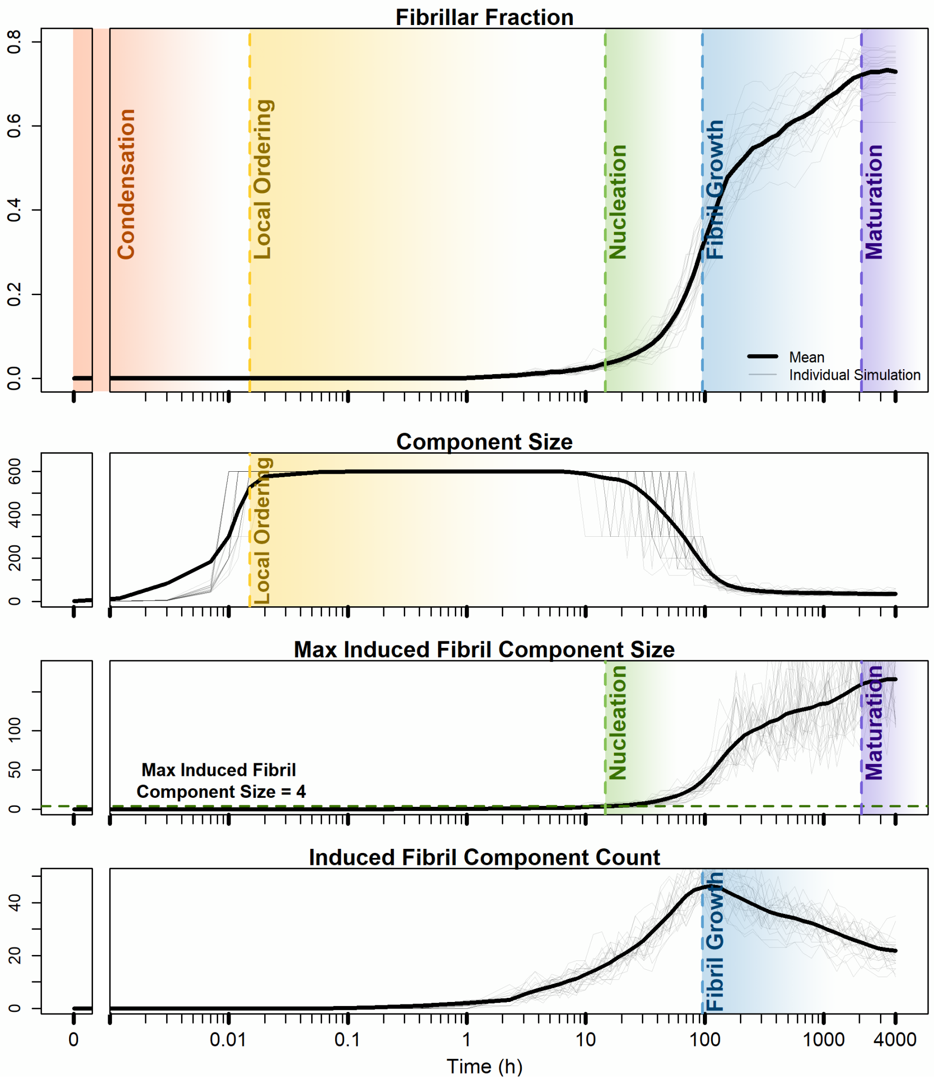

Figure S4: Characteristic aggregation dynamics for the 3-prism amyloid fibril network topology.

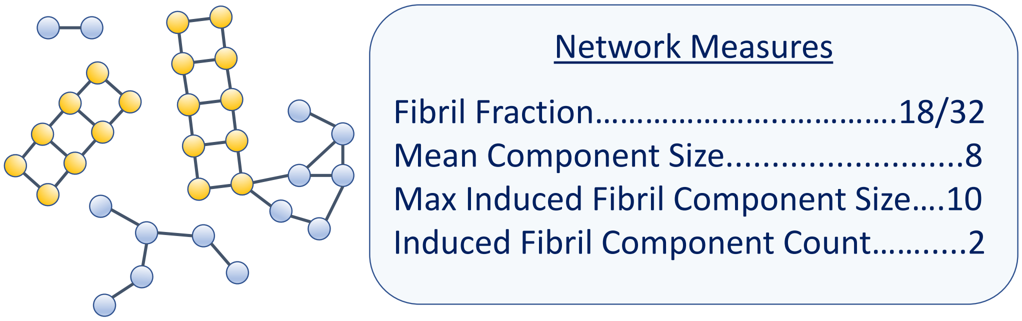

Figure S5: Heuristic example of four key network measures used to define fibril growth epochs, demonstrated on a 32 node graph featuring 2-ribbon formation (yellow). Fibril fraction is the number of nodes comprising 2-ribbon induced subgraphs divided by the total number of nodes in the system. There are 4 components present of sizes 2, 8, 6, and 16, leading to a mean component size of 8. The largest induced fibril component is the 10 node fibril embedded in the largest component shown. Finally, there are two components present that possess induced fibril structure, making the induced fibril component count equal to 2.

References

[1] Grazioli, G., Yu, Y., Unhelkar, M. H., Martin, R. W., Butts, C. T. Network-based classification and modeling of amyloid fibrils. *The Journal of Physical Chemistry B*, **123**, 5452–5462 (2019).

[2] Lusher, D., Koskinen, J., Robins, G. *Exponential random graph models for social networks: Theory, methods, and applications*; Cambridge University Press: Cambridge (2012).
